# Imaging features of multimodal ultrasound and dynamic contrast-enhanced MRI in breast granular cell tumor: A case report

**DOI:** 10.1016/j.radcr.2026.04.075

**Published:** 2026-05-20

**Authors:** Hui Li, Yani Chao, Maolin Wu

**Affiliations:** a Department of Ultrasound, Tianshui First People's Hospital, Tianshui, Gansu, China; b Department of Ultrasound, Gansu University of Chinese Medicine First School of Clinical Medical, Lanzhou, Gansu, China

**Keywords:** Granular cell tumor of the breast, Multimodal ultrasound, DCE-MRI

## Abstract

Granular cell tumor of the breast (GCTB) is a rare benign lesion that constitutes a well-recognized diagnostic pitfall owing to its potential to simulate malignant breast neoplasms on imaging. We present a 50-year-old woman with a palpable, painless breast mass demonstrating relatively well-defined borders on imaging. Multimodal ultrasound was highly suspicious for malignancy, whereas dynamic contrast-enhanced magnetic resonance imaging (DCE-MRI) favored a benign process. Core needle biopsy with immunohistochemistry established the diagnosis of benign GCTB. This case highlights that GCTB may closely mimic breast cancer on ultrasound, which can lead to misdiagnosis and unnecessary overtreatment. For radiologists, recognition of this diagnostic pitfall is critical: ultrasound features suggestive of malignancy do not reliably exclude GCTB. A multimodal imaging approach, supplemented by targeted core needle biopsy and immunohistochemistry, is essential to avoid misinterpretation and establish the correct diagnosis.

## Introduction

GCTB is a rare benign neoplasm originating from Schwann cells, accounting for approximately 0.5% of all breast tumors [1,2], it typically arises within the intralobular stroma of the breast, often in the upper inner quadrant, a location consistent with the distribution of the cortical branch of the supraclavicular nerve [3]. Despites its benign nature, GCTB often presents with aggressive clinical and radiological features, such as a painless, firm mass with irregular and ill-defined borders [4]. These characteristics make it clinically challenging to differentiate from malignant breast carcinoma, frequently leading to unnecessary biopsies or surgical rescetions.

With advancements in imaging technology, multimodal ultrasound and DCE-MRI have become crucial tools for the differential diagnosis of breast masses [5]. However, due to the rarity of GCTB, comprehensive studies on the combined imaging features of this tumor, especially when findings from different imaging modalities are discordant, remain limited [6]. Therefore, a thorough analysis of the imaging characteristics of GCTB holds great significance for improving diagnostic accuracy and preventing misdiagnosis.

This paper aims to report a case of a pathologically confirmed granular cell tumor of the breast. We will provide a detailed description of this imaging findings on multimodal ultrasound, including conventional grayscale, color Doppler, microfluidic imaging, and shear-wave elastography, along with the results from dynamic contrast-enhanced magnetic resonance imaging. By presenting this case, we hope to explore the comprehensive imaging features of GCTB and provides valuable reference for the differential diagnosis of breast masses in clinical practice.

## Case presentation

**Fig. 1:**
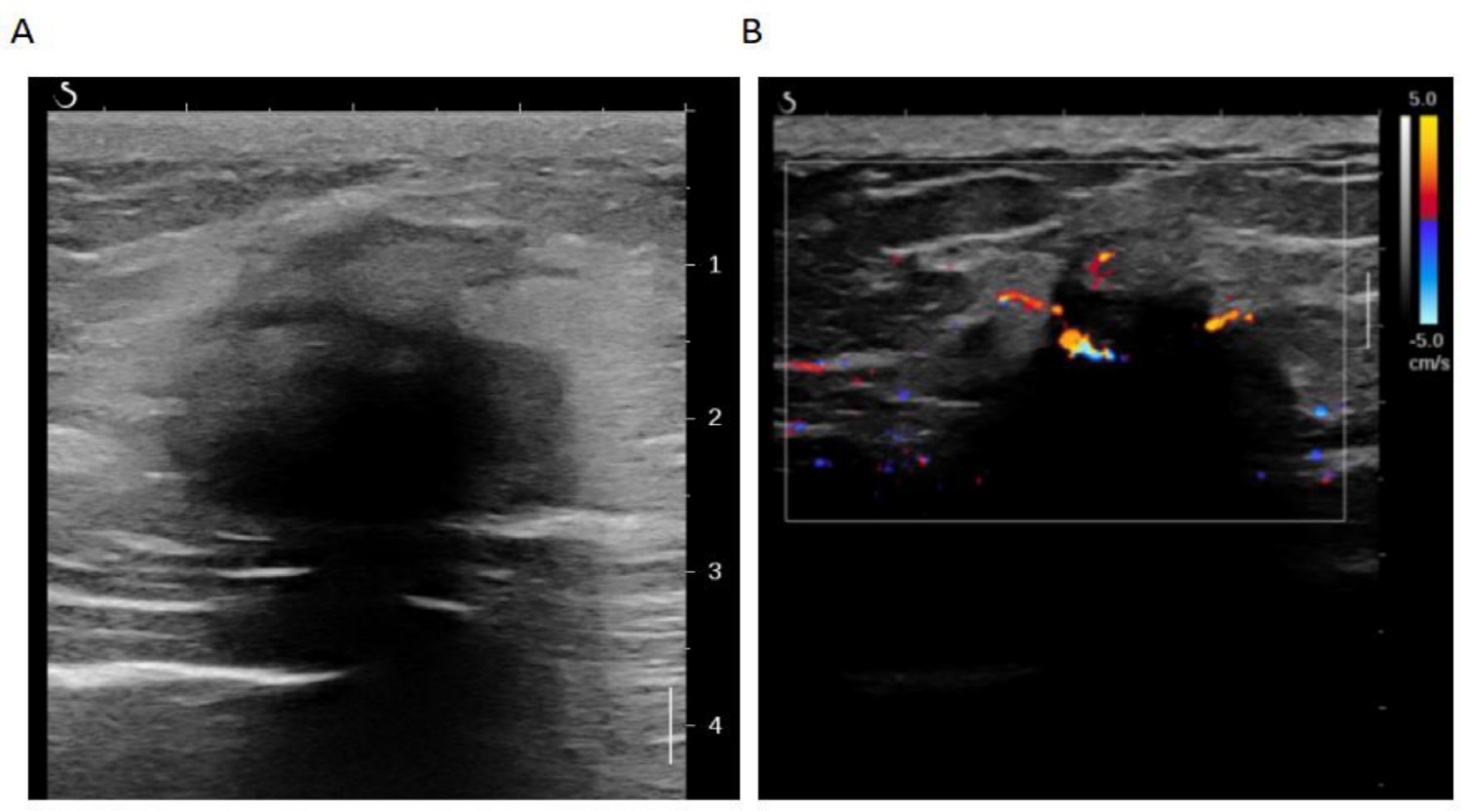
(A) 2D US: Left breast hypoechoic mass, unclear border, irregular shape, BI-RADS 4b. (B) CDFI: Minimal short rod-like blood flow around the mass.

**Fig. 2:**
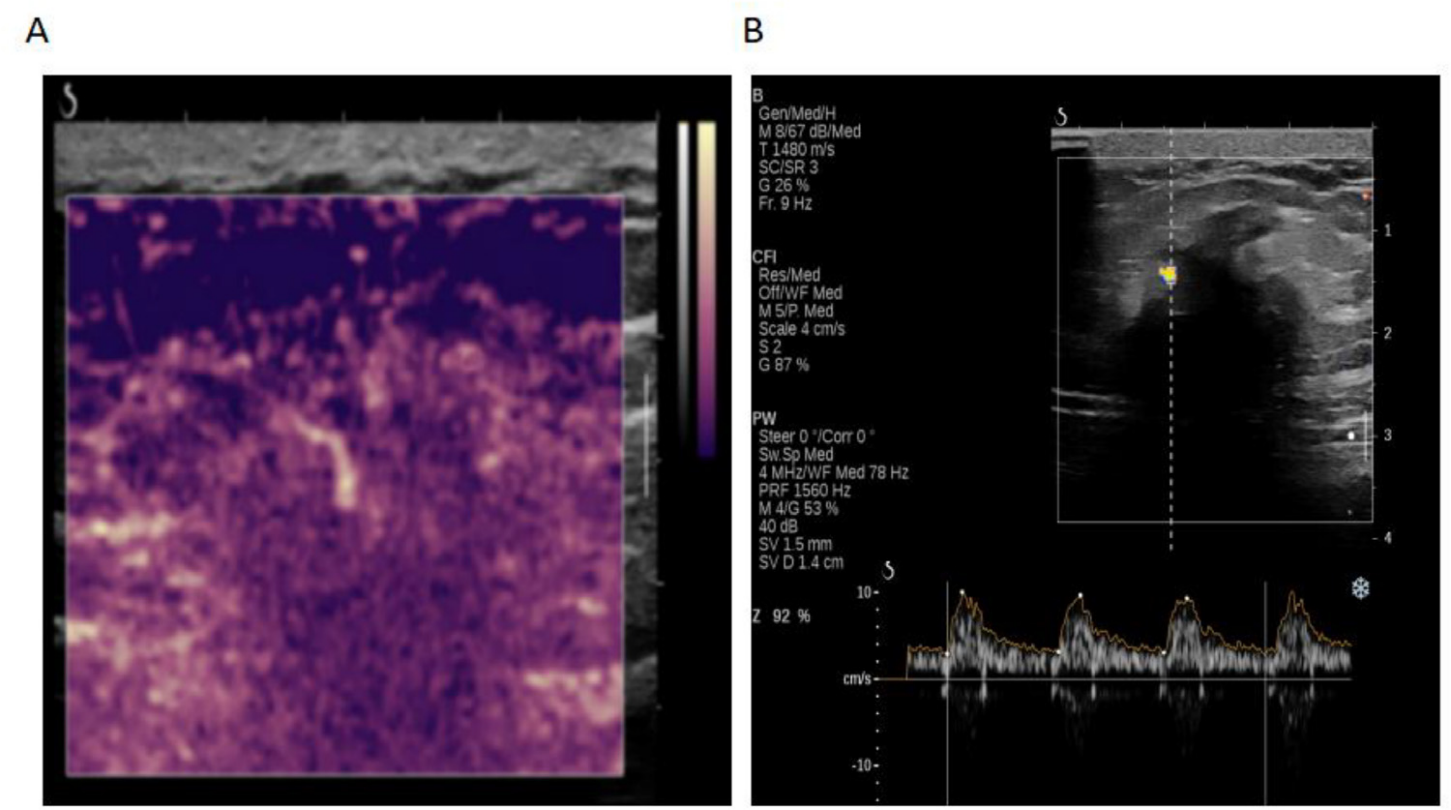
(A) Ultrasound microvascular imaging: Low-velocity microflow within the mass. (B) Pulsed Doppler: Low-velocity arterial-like flow spectrum.

A 50-year-old woman presented with a 1-year history of a painless, palpable mass in her left breast. Initial 2D gray-scale ultrasound revealed a hypoechoic mass with irregular shape and angular margins, measuring 30 × 26 mm, located at the 10 o'clock position (45 mm from the nipple and 13 mm from the skin) (Fig. 1A). The lesion demonstrated an ill-defined, spiculated border with posterior acoustic shadowing—features classified as BI-RADS category 4b (moderate suspicion for malignancy, estimated cancer risk 10%-50%). Color Doppler flow imaging showed a small amount of peripheral, short rod-shaped blood flow signal (Fig. 1B), while ultrasound microfluidic imaging demonstrated very low-velocity microvascular flow within the mass (Fig. 2A) with pulsed Doppler showing a low-velocity arterial-like spectrum (Fig. 2B).

**Fig. 3:**
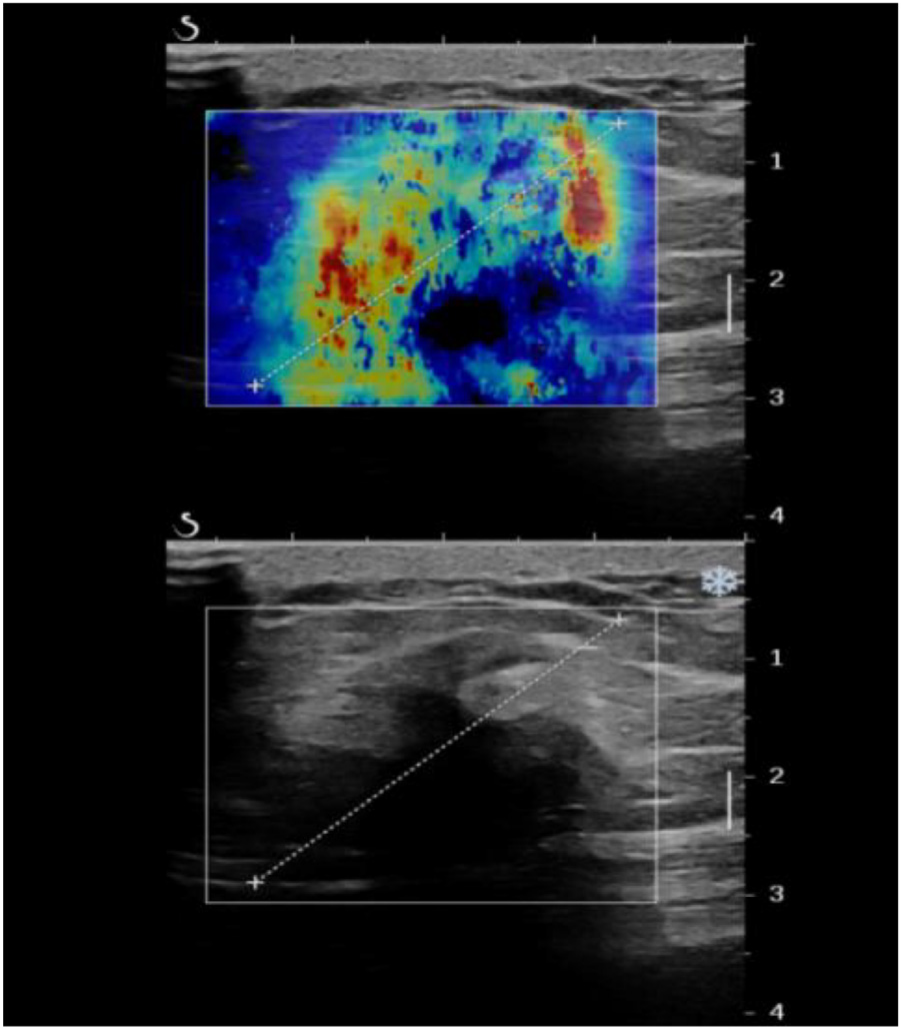
SWE: Peripheral yellow-red with central shear wave loss, hard ring sign; suggestive of malignant mass.

**Fig. 4:**
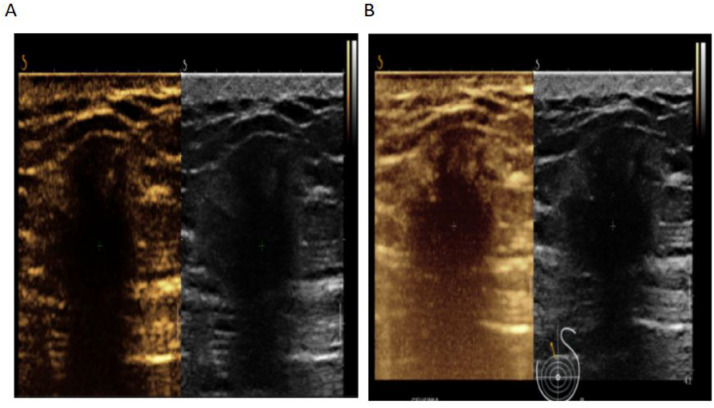
CEUS findings. (A) Arterial phase: inhomogeneous peripheral circumferential enhancement, central non-enhancement. (B) Late arterial phase: persistent peripheral microbubble retention.

Shear-wave elastography indicated a predominantly yellow and red pattern with some shear wave loss, suggesting the mass was hard and has a high suspicious for malignancy (Fig. 3). The patient underwent contrast-enhanced ultrasound with an intravenous injection of SonoVue 2.4 mL followed by a 10 mL saline flush (0.9% NaCl). Subsequent contrast-enhanced ultrasound (CEUS) showed that the contrast agent began to enhance in the peripheral portion of the mass 16 seconds after injection, with inhomogeneous circumferential hyperenhancement, while most of the central portion showed no contrast entry (Fig. 4A). Microbubbles remained visible in the periphery of the mass at 1 minute post-injection (Fig. 4B).

**Fig. 5:**
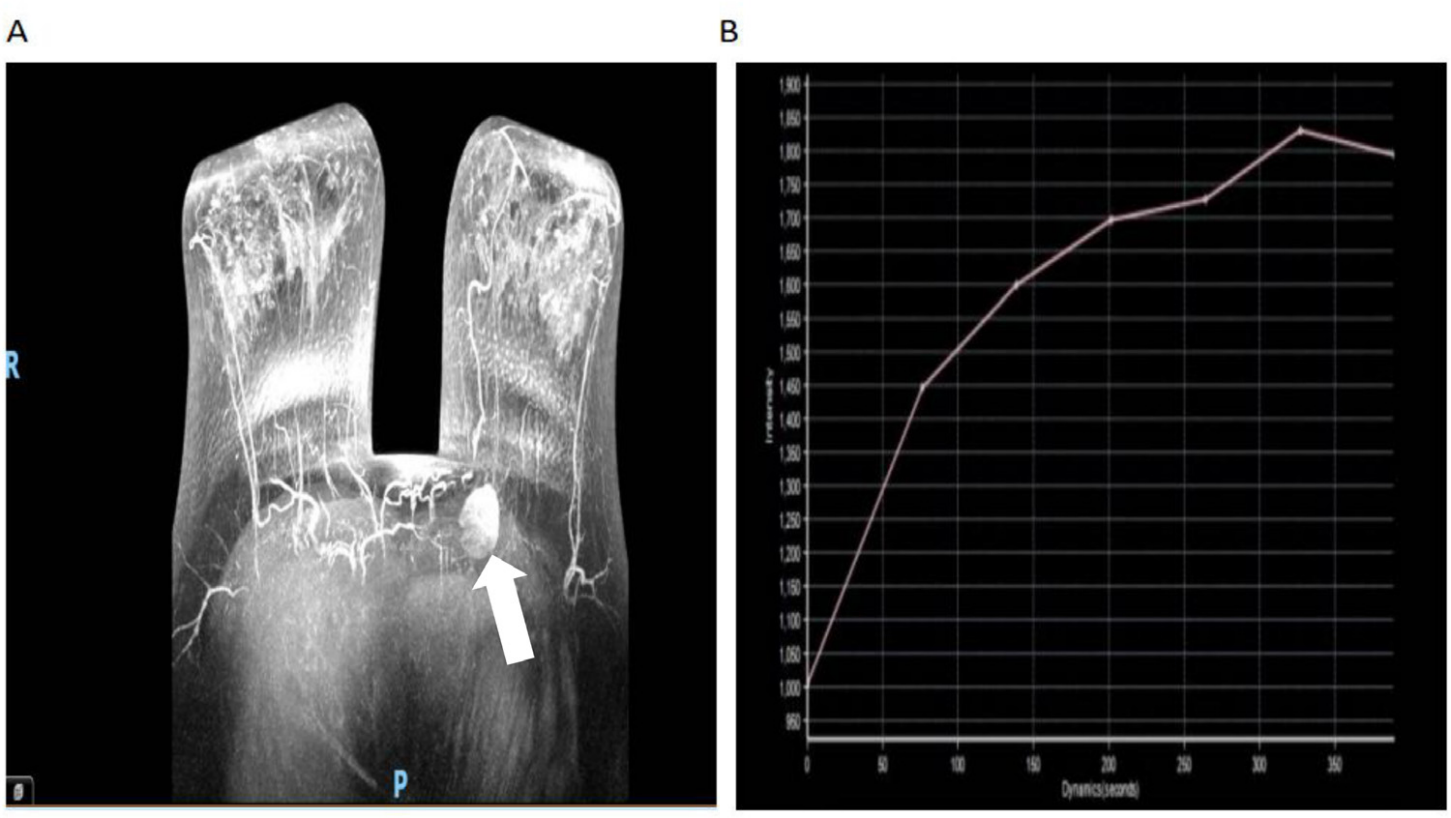
(A) DCE-MR: Left breast mass with heterogeneous enhancement (axial view, mass location indicated by white arrow). (B) Time-signal-intensity curve: Type I (mildly progressive, inflow pattern).

**Fig. 6:**
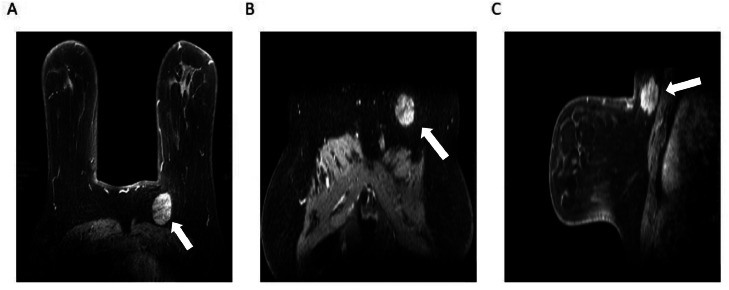
Contrast-enhanced fat-suppressed MRI: Left breast mass shows heterogeneous enhancement (Panel A: axial view, Panel B: coronal view, Panel C: lateral view, mass location indicated by white arrow).

Despite these suspicious ultrasound findings, a DCE-MRI scan revealed an approximately 2.8 cm x 2.5 cm x 2.7 cm mass in the upper inner quadrant of the left breast with significant inhomogeneous enhancement and linear non-enhancing segments (Fig. 5A and Fig. 6). Multiple-point measurements of the time-signal intensity curve show a benign inflow pattern with no significant 'washout' phenomenon, and the early enhancement rate is approximately 65% (Fig. 5B). Serum tumor markers were within the normal range (CEA 2.36 ng/mL; CA153 8.36 U/mL).

**Fig. 7:**
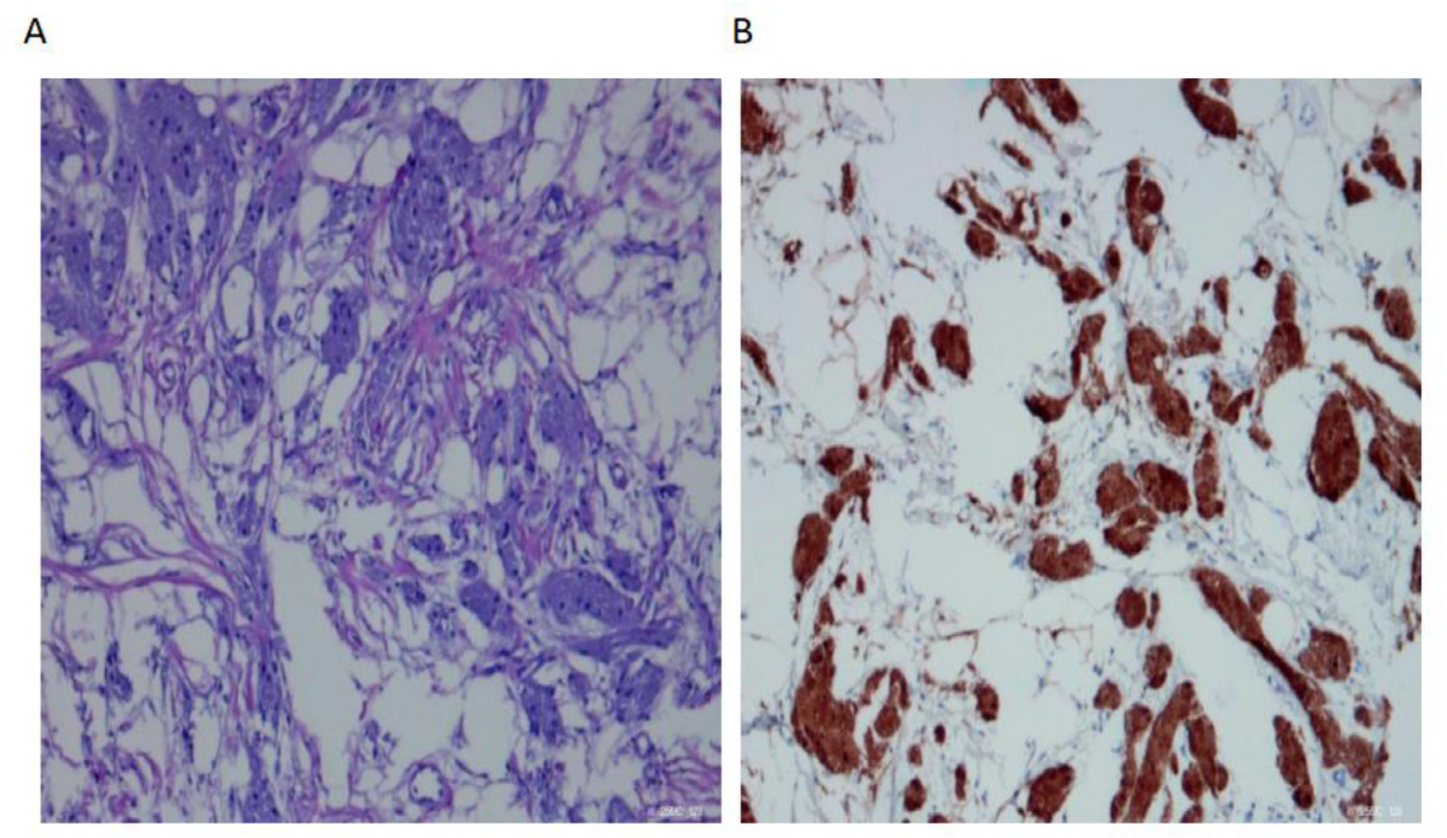
Pathological findings. (A) HE × 200: Tumor cells arranged in nests and broad bands with abundant granular cytoplasm.(B) IHC × 200: Tumor cells positive for S-100 protein.

After informed consent, the patient initially underwent ultrasound-guided core needle biopsy, which histopathologically confirmed a benign breast granular cell tumor. Wide local excision of the breast mass was subsequently performed, and the excisional biopsy further verified the diagnosis. Histopathological examination revealed that the tumor cells arranged in nests and broad bands with abundant granular cytoplasm and small, uniform nuclei (Fig. 7A). Immunohistochemical staining was positive for S100, Ki-67 (1%), TFE3, CR, Vim, and CD68, and negative for CK-pan, ER, GCDPF-15, SMA, and other markers (Fig. 7B). The patient recovered well after surgery with no signs of recurrence at the 6-month follow-up.

## Discussion

Clinically, GCTB often presents as a firm, painless mass, although a subset of patients may experience discomfort, itching, or skin changes such as retraction, thickening, or dimpling, as well as enlarged reactive lymph nodes.

On ultrasonography, granular cell tumors (GCTs) of the breast frequently demonstrate malignant-mimicking features that create significant diagnostic challenges. According to existing literature [[7], [8], [9]], these tumors may commonly present with marked posterior acoustic shadowing due to dense stromal fibrosis, resembling malignant tumor attenuation. On elastography, GCT shows markedly increased stiffness comparable to invasive carcinoma, further blurring benign-malignant differentiation. Color Doppler frequently detects internal or peripheral vascularity consistent with malignant neoplasms, making preoperative distinction extremely challenging without histopathology.

On mammographic examination, GCTs typically present as irregular, high-density masses with spiculated or indistinct margins, features that are classically associated with malignancy and often result in BI-RADS category 4 or 5 classifications [10]. However, a limitation of the present case is the absence of mammographic imaging, which precludes a complete radiological assessment and direct correlation with the typical imaging features described in the literature.

For breast MRI, GCTs are typically isointense or mildly hypointense on T2-weighted images compared to breast parenchyma, contrasting with the hyperintense signal usually seen in invasive ductal carcinoma [11]. After contrast agent administration, most GCTs exhibit progressive persistent enhancement (Type I kinetic curve), a pattern that is highly suggestive of a benign nature [10]. This gradual enhancement is a reflection of slow interstitial perfusion without rapid washout, which distinguishes GCTs from invasive breast cancer that typically shows a Type III (washout) kinetic curve. The presence of a progressive enhancement curve supports a benign biological behavior, even when the morphological features are suspicious.

Due to this wide range of appearances, distinguishing GCT from malignant breast tumors based on clinical and imaging findings alone can be challenging [12]. A definitive diagnosis of GCTB relies on histopathological examination, which typically reveals cells with abundant granular cytoplasm that stain positively for S100 protein, CD68, and neuron-specific enolase (NSE) on immunohistochemical analysis [13].

Considering the high incidence of imaging overlap and false-suspicious features between GCTs and breast cancer, biopsy is imperative in cases of radiological inconsistency or high clinical suspicion. The indications for immediate biopsy include: (1) discordant findings between ultrasound and MRI, such as 1 modality suggesting malignancy while the other demonstrates benign characteristics [8]; (2) BI-RADS classification of 4 or higher regardless of the imaging modality [11]; (3) Palpable firm mass accompanied by suspicious ultrasound elastometry findings or mammographic structural distortion [8]; (4) progressive lesion enlargement or clinical manifestations raising concern for breast cancer [14]; (5) any imaging presentation that falls into established diagnostic pitfalls of GCT mimicry [14] (Table 1).

**Table 1:** Multimodality characteristics of breast granular cell tumors.

Modality	Core features	Discriminatory value
Ultrasonography	Irregular margins; marked posterior acoustic shadowing; increased stiffness on elastography; hypervascularity	Malignant-mimicking morphology; shadowing and stiffness may mislead toward BI-RADS 4-5 classification
MRI	T2 iso-/hypointense (vs. T2 hyperintense in invasive carcinoma); progressive persistent enhancement (Type I curve)	T2 signal + Type I curve favor benignity despite suspicious morphology
Histopathology	Tumor cells arranged in nests or fascicles with abundant eosinophilic cytoplasm; S-100 strong positivity	S-100 confirms diagnosis

Surgical excision with clear margins remains the standard treatment, and adequate resection is critical to minimize local recurrence. The importance of margin status has been emphasized across anatomical sites: historical data from large soft tissue GCT series indicate that incomplete surgical resection substantially elevates recurrence risk—from approximately 2%-8% following negative margin excision to over 20% when resection margins are compromised [15]. However, breast-specific investigations suggest a more favorable biological behavior for mammary GCTs. In a dedicated clinicopathologic analysis with extended observation period, no disease recurrence was documented among 13 patients despite the presence of positive or close (<1 mm) surgical margins, challenging the conventional margin-driven prognostic paradigm [16]. Nevertheless, given the potential for rare malignant transformation and the difficulty of preoperative differentiation, complete excision with clear margins remains the recommended standard [17].

Following excision, patients require clinical and imaging surveillance to detect recurrence or metachronous lesions. While benign GCTs rarely metastasize, sporadic cases of metastasis from histologically benign primaries have been documented, mandating long-term follow-up [18].

## Conclusion

This case highlights that GCTB may demonstrate suspicious malignant features on imaging, which can create diagnostic overlap with breast cancer and pose challenges in differentiation. We suggest that a comprehensive multimodality imaging evaluation-encompassing mammography, multimodal ultrasound, and dynamic contrast-enhanced magnetic resonance imaging-supplemented by core needle biopsy, may provide useful diagnostic information and help reduce the risk of misdiagnosis before definitive surgical intervention.

## Patient consent

Written informed consent was obtained from the patient for the publication of this case report, including all clinical details, images, and any other identifiable information presented in this article.
